# Discovery and engineering of an endophytic *Pseudomonas* strain from *Taxus chinensis* for efficient production of zeaxanthin diglucoside

**DOI:** 10.1186/s13036-019-0196-x

**Published:** 2019-08-01

**Authors:** Ozkan Fidan, Jixun Zhan

**Affiliations:** 1 Department of Biological Engineering, Utah State University, 4105 Old Main Hill, Logan, UT 84322-4105 USA; 2 TCM and Ethnomedicine Innovation & Development Laboratory, School of Pharmacy, Hunan University of Chinese Medicine, Changsha, 410208 Hunan China

**Keywords:** Carotenoids, *Pseudomonas*, Endophyte, *Taxus chinensis*, Heterologous expression, Zeaxanthin diglucoside

## Abstract

**Background:**

Endophytic microorganisms are a rich source of bioactive natural products. They are considered as promising biofertilizers and biocontrol agents due to their growth-promoting interactions with the host plants and their bioactive secondary metabolites that can help manage plant pathogens. Identification of new endophytes may lead to the discovery of novel molecules or provide new strains for production of valuable compounds.

**Results:**

In this study, we isolated an endophytic bacterium from the leaves of *Taxus chinensis*, which was identified as *Pseudomonas* sp. 102515 based on the 16S rRNA gene sequence and physiological characteristics. Analysis of its secondary metabolites revealed that this endophytic strain produces a major product zeaxanthin diglucoside, a promising antioxidant natural product that belongs to the family of carotenoids. A carotenoid (*Pscrt*) biosynthetic gene cluster was amplified from this strain, and the functions of PsCrtI and PsCrtY in the biosynthesis of zeaxanthin diglucoside were characterized in *Escherichia coli* BL21(DE3). The entire *Pscrt* biosynthetic gene cluster was successfully reconstituted in *E. coli* BL21(DE3) and *Pseudomonas putida* KT2440. The production of zeaxanthin diglucoside in *Pseudomonas* sp. 102515 was improved through the optimization of fermentation conditions such as medium, cultivation temperature and culture time. The highest yield under the optimized conditions reached 206 mg/L. The engineered strain of *P. putida* KT2440 produced zeaxanthin diglucoside at 121 mg/L in SOC medium supplemented with 0.5% glycerol at 18 °C, while the yield of zeaxanthin diglucoside in *E. coli* BL21(DE3) was only 2 mg/L. To further enhance the production, we introduced an expression plasmid harboring the *Pscrt* biosynthetic gene cluster into *Pseudomonas* sp. 102515. The yield in this engineered strain reached 380 mg/L, 85% higher than the wild type. Through PCR, we also discovered the presence of a turnerbactin biosynthetic gene cluster in *Pseudomonas* sp. 102515. Because turnerbactin is involved in nitrogen fixation, this endophytic strain might have a role in promoting growth of the host plant.

**Conclusions:**

We isolated and identified an endophytic strain of *Pseudomonas* from *T. chinensis*. A zeaxanthin diglucoside biosynthetic gene cluster was discovered and characterized in this bacterium. Through fermentation and genetic engineering, the engineered strain produced zeaxanthin diglucoside at 380 ± 12 mg/L, representing a promising strain for the production of this antioxidant natural product. Additionally, *Pseudomonas* sp. 102515 might also be utilized as a plant-promoting strain for agricultural applications.

**Electronic supplementary material:**

The online version of this article (10.1186/s13036-019-0196-x) contains supplementary material, which is available to authorized users.

## Background

Nature provides a huge repertoire of structurally and functionally diverse bioactive molecules. As such, natural products are a major source of new drugs. Microorganisms are known to produce many pharmaceuticals such as lovastatin (anti-cholesterol), penicillin (antibacterial) and vancomycin (antibacterial). There is an estimated 99.999% of microbial species left undiscovered in the world. Therefore, microorganisms remain an underexplored source of bioactive natural products. In particular, endophytic microorganisms hold huge potential for the discovery of natural products with pharmaceutical importance [1]. Endophytes, microorganisms (bacteria and fungi) that live in the tissues of living plants without causing any apparent disease symptoms in the host, are potential sources of novel natural products with applications in medicine, agriculture, and industry [2, 3]. They have been isolated from medicinal plants, weeds, as well as ornamental and fruit trees [1]. Endophytes enter plants through the roots and the aerial portions of plants, such as leaves, flowers, stems and cotyledons [4]. Upon entering the host, they reside within cells, the intercellular spaces or the vascular system [5]. Some endophytes exhibit plant growth-promoting effects and protect plants from biotic and abiotic stresses under different environmental conditions. Thus, they are considered as endosymbiotic microorganisms with potential agricultural applications as biofertilizers and biocontrol agents [5–8].

Another important aspect is that the endophytes are known to produce a wide variety of natural products. For example, ecomycins produced by *Pseudomonas viridiflava* are a family of novel lipopeptides and consist of some unusual amino acids including homoserine and β-hydroxy aspartic acid [9]. Another example is pseudomycins, which represent a group of antifungal peptide compounds isolated from *Pseudomonas syringae*, a plant-associated bacterium [10]. The new species can sometimes lead to the rediscovery of known natural products as happened in the example of paclitaxel (Taxol®). Taxol, the world’s first billion-dollar anticancer drug, was isolated from the Pacific yew tree, *Taxus wallachiana*. Interestingly, endophytic fungus (*Pestalotiopsis microspora*) isolated from this plant was reported to produce paclitaxel [11], representing a potential microbial host for the production of this pharmaceutically important molecule.

Carotenoids are isoprenoid pigments that are widely seen in nature. They are produced by all known phototrophic organisms and some non-phototrophic fungi, bacteria and archaea [12]. Carotenoids exhibit diverse biological functions in different organisms that either produce or consume carotenoids. They serve as accessory pigments in the light harvesting center of phototrophic organisms [13]. The primary roles of carotenoids in non-phototrophic organisms are membrane stability and relief of oxidative stress [14]. Additionally, carotenoids have been used in various applications, ranging from food colorants and feed supplements to nutritional and cosmetics purposes [15]. This family of natural products has shown a variety of biological activities, such as antioxidant, anticancer, and anti-inflammatory properties. They are also used as precursors for the synthesis of vitamin A and as nutritional factors for the prevention of chronic diseases [16, 17]. Due to the negative impacts of synthetic coloring agents on human health (e.g., toxicity, hyperallergenicity, and carcinogenicity) and the increase in the consumer demand for natural and health-promoting food ingredients, the utilization of carotenoids as colorants and supplements is rising in food, cosmetics, nutraceutical and pharmaceutical industries, with an expected global market value of $1.8 billion in 2019 [18, 19].

**Fig. 1:**
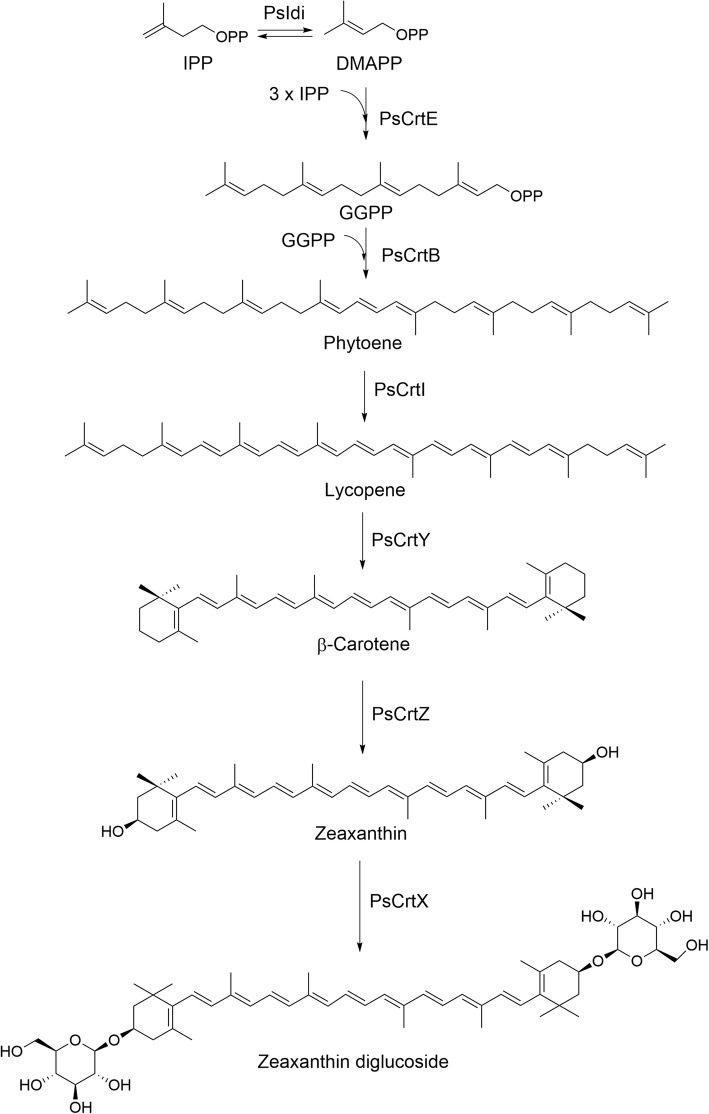
Proposed biosynthetic pathway of zeaxanthin diglucoside in *Pseudomonas* sp. 102515. The involved enzymes include isopentenyl-diphosphate delta-isomerase (PsIdi), geranylgeranyl pyrophosphate synthetase (PsCrtE), phytoene synthase (PsCrtB), lycopene synthase (PSCrtI), lycopene cyclase (PsCrtY), β-carotene hydroxylase (PsCrtZ), and zeaxanthin glucosyltransferase (PsCrtX)

More than 750 different natural carotenoids have been identified. These molecules share a linear, conjugated chromophore backbone and a common biosynthetic pathway (Fig. 1). Carotenoid biosynthesis typically begins with the isomerization of isopenthyl diphosphate (IPP) from the mevalonate pathway to yield dimethylallyl diphosphate (DMAPP), catalyzed by the IPP isomerase (Idi). Geranyl diphosphate (GPP) is synthesized through the head-to-tail condensation of IPP and DMAPP. Addition of IPP to GPP generates farnesyl pyrophosphate (FPP) formation and a further IPP molecule yields geranylgeranyl diphosphate (GGPP) by geranylgeranyl diphosphate synthase (CrtE). Phytoene synthase (CrtB) catalyzes the head-to-head condensation of two GGPP to phytoene. Phytoene desaturase (CrtI) extends the double bond conjugation of phytoene to generate lycopene. Terminal β-cyclization catalyzed by lycopene β-cyclase (CrtY) results in the formation of β-carotene, which is subsequently hydroxylated by β-carotene hydroxylase (CrtZ), yielding zeaxanthin. Zeaxanthin can be further modified by different tailoring enzymes to yield a variety of carotenoids. For example, it can be glycosylated by the glycosyltransferase (CrtX) to generate zeaxanthin diglucoside [20–23].

In this study, our primary purpose was to isolate endophytic bacteria with the purpose of discovering new natural products and/or new effective means to produce carotenoids. We isolated an endophytic *Pseudomonas* strain, named *Pseudomonas* sp. 102515, from the leaves of the yew tree, which was found to produce zeaxanthin diglucoside as the major product. Based on the genome of *P. psychrotolerans* PRS08–11306 (the closest relative to the endophytic strain) as reference, we were able to amplify a carotenoid (*Pscrt*) biosynthetic gene cluster from *Pseudomonas* sp. 102515. Two genes in this gene cluster were functionally characterized. The entire *Pscrt* gene cluster was successfully expressed in two heterologous hosts, *Escherichia coli* BL21(DE3) and *Pseudomonas putida* KT2440. Additionally, the production of zeaxanthin diglucoside in *Pseudomonas* sp. 102515 was significantly improved by optimizing the fermentation conditions, with a yield of 206 ± 6 mg/L. This endophyte was further engineered by introducing an expression plasmid harboring the *Pscrt* gene cluster. The yield of zeaxanthin diglucoside by the engineered strain reached 380 ± 12 mg/L, 85% higher than the wild type strain. This strain represents a promising host for the production of zeaxanthin diglucoside.

## Results

### Identification and phylogenetic analysis of an endophytic bacterium from *Taxus chinensis*

**Fig. 2:**
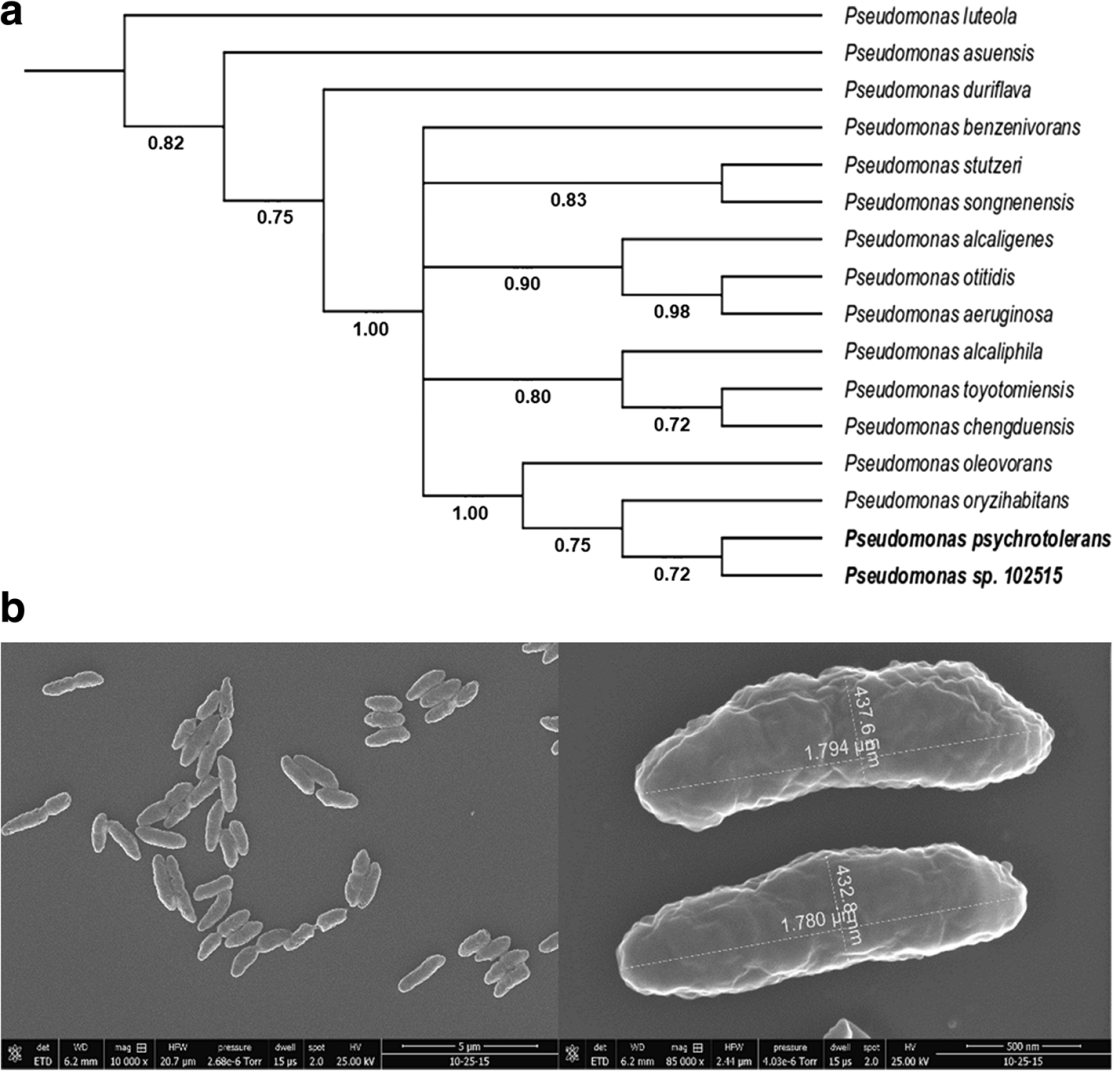
Identification of *Pseudomonas* sp. 102515. (**a**) A phylogenetic tree constructed based on the 16S rRNA sequences of *Pseudomonas* strains from BLASTn analysis. It estimates the relationship between strain 102515 and other *Pseudomonas* strains that shared the highest 16S rRNA gene sequence similarities. Numbers at the nodes indicate the normalized percentages of occurrence in 100 bootstrapped trees, and only values greater than 0.5 are shown. Sequences of reference species were obtained from GenBank, including *P. luteola* (NR_114215), *P. asuensis* (NR_136445), *P. duriflava* (NR_044390), *P. benzenivorans* (NR_116904), *P.stutzeri* (NR_041715), *P. songnenensis* (NR_148295), *P. alcaligenes* (NR_113646), *P. otitidis* (NR_043289), *P. aeruginosa* (NR_117678), *P. alcaliphila* (NR_114072), *P. toyotomiensis* (NR_112808), *P. chengduensis* (NR_125523), *P.oleovorans* (NR_043423), *P. oryzihabitans* (NR_114041), and *P. psychrotolerans* (NR_042191.1). (**b**) SEM micrograph of *Pseudomonas* sp. 102515. The bacterium was fixed with 2% glutaraldehyde in 0.1% HEPES buffer overnight. The samples were subjected to alcohol series dehydration (50–100% ethanol) and then chemically dried using hexamethyldisilazane

We isolated an endophytic bacterium from the leaves of *Taxus chinensis*, whose colonies showed a bright yellow color. To identify this strain, we amplified the 16S ribosomal RNA (rRNA) gene fragment by PCR using a set of universal primers. The gene sequence of this fragment was subjected to BLAST analysis, with the 16S rRNA sequences (bacteria and archaea) as the reference database. The BLASTn analysis revealed that there are three *Pseudomonas* strains with 99% identity and coverage. Some other *Pseudomonas* strains (*Pseudomonas stutzeri*, *Pseudomonas indoloxydans*, *Pseudomonas luteola,* etc.) also had 99% coverage but are 96% identical to our endophytic isolate. Using an online platform (https://itol.embl.de/), we constructed a phylogenetic tree by the neighbor-joining method (Fig. 2a). The phylogenetic tree indicated that *P. psychrotolerans* is the closest relative to our isolate. *P. oryzihabitans* and *P. oleovorans* are also closely related to this endophytic strain. We also looked into the physiology of our isolate under a scanning electron microscope (SEM) (Fig. 2b) and confirmed that it is a rod-shaped bacterium. Additionally, it is a non-spore-forming and a yellow-pigment-producing bacterium. The closest relative, *P. psychrotolerans*, was also reported as a yellow-pigmented bacterium [24]. All these physiological characteristics and genetic analysis indicated that this endophyte is a *Pseudomonas* strain, which was named as *Pseudomonas* sp. 102515.

### Amplification and analysis of a zeaxanthin diglucoside biosynthetic gene cluster from *Pseudomonas* sp. 102515

As described above, *P. psychrotolerans* is the closest relative strain to *Pseudomonas* sp. 102515. The genome of this strain is available in GenBank under the accession number, NZ_CP018758. We analyzed the genome of *P. psychrotolerans* with the help of the AntiSMASH online genome analysis platform. Four major natural product biosynthetic gene clusters were identified. The first one is a nonribosomal peptide synthetase (NRPS)-type gene cluster with 50% similarity to the known taiwachelin biosynthetic gene cluster. A turnerbactin (*tnb*) biosynthetic gene cluster was also found to be located in this NRPS gene cluster. Turnerbactin was reported to have an important role in the plant-microbe interactions [25]. In order to find out whether a *tnb* gene cluster is present in *Pseudomonas* sp. 102515, we amplified the *tnbA* gene from its genomic DNA using a pair of primers designed based on the genome of *P. psychrotolerans*. The sequence of *tnbA* was provided in the Supporting Information (Sequence S1). The second gene cluster is an arylpolyene (APE)-type biosynthetic gene cluster from which 40% of genes show a similarity to the genes from the characterized APE biosynthetic gene cluster. APE gene clusters are widely distributed in gram-negative bacteria, including *Pseudomonas* strains [26]. In addition to another siderophore biosynthetic gene cluster, genome analysis revealed that *P. psychrotolerans* has a carotenoid (*crt*) biosynthetic gene cluster as expected from its carotenoid production capability. The genes from this *crt* gene cluster is similar to those found in the *crt* gene cluster from *Pantoea agglomerans*, which contains *crtE*, *idi*, *crtX*, *crtY*, *crtI*, *crtB* and *crtZ* [27].

**Fig. 3:**
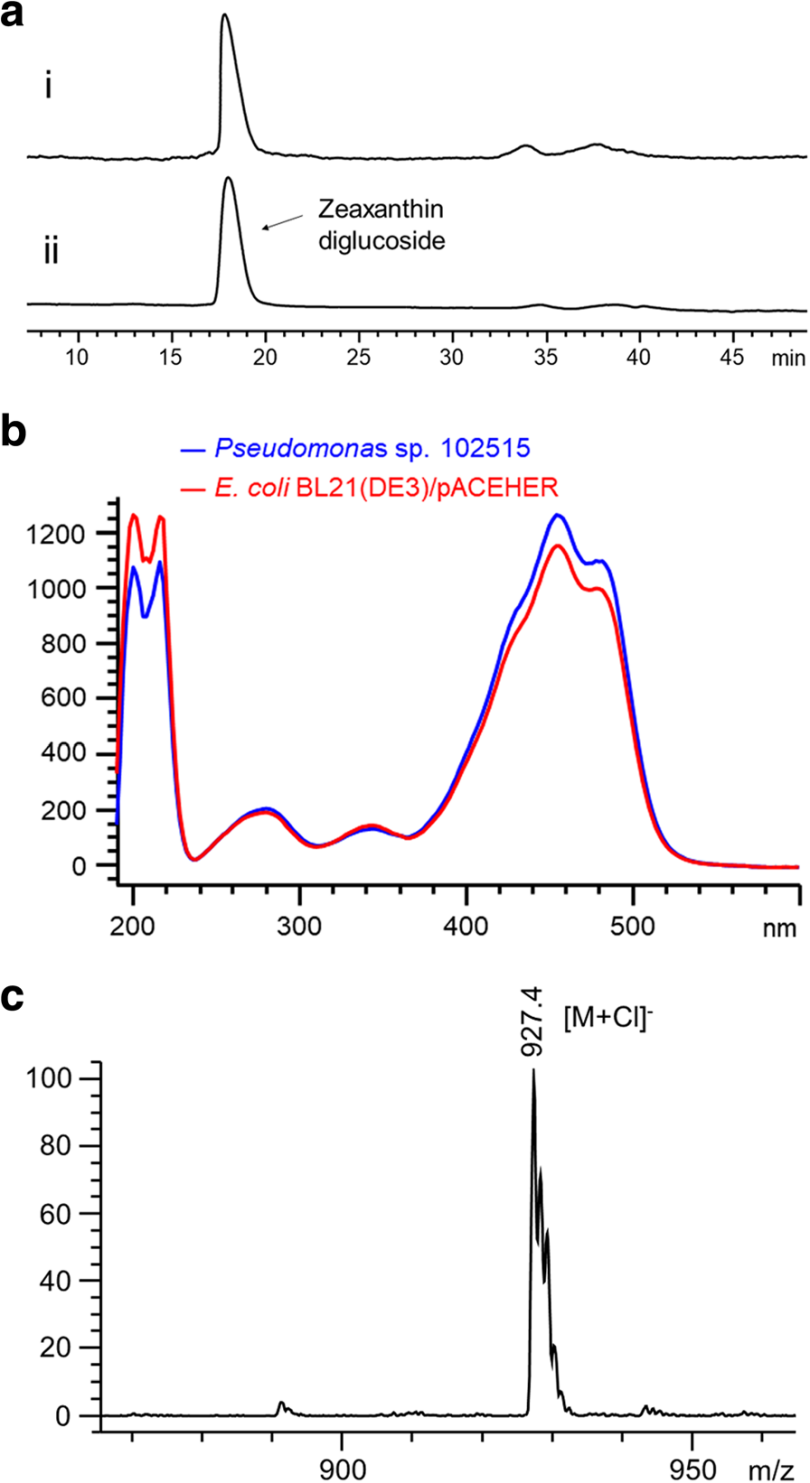
Production of zeaxanthin diglucoside by *Pseudomonas* sp. 102515. (**a**) HPLC analysis (460 nm) of zeaxanthin diglucoside production by *Pseudomonas* sp. 102515. (i) *Pseudomonas* sp. 102515, (ii) *E. coli* BL21(DE3)/pAC-EHER. (**b**) Comparison of the UV spectra of the major product of *Pseudomonas* sp. 102515 and *E. coli* BL21(DE3)/pAC-EHER. (**c**) ESI-MS(−) spectrum of the major product of *Pseudomonas* sp. 102515

To find out what yellow compound *Pseudomonas* sp. 102515 produced, we extracted the cells with methanol/chloroform (2:1, v/v) and analyzed the products by LC-MS. The extract of *Pseudomonas* sp. 102515 showed a major peak at 18.2 min (Fig. 3a). Based on its UV and MS spectra (Fig. 3b and c), this compound was characterized as zeaxanthin diglucoside. Furthermore, this peak has the same retention time and UV spectrum as zeaxanthin diglucoside produced by *E. coli* BL21(DE3)/pAC-EHER [28]. Therefore, we confirmed that the yellow pigment from this endophyte is zeaxanthin diglucoside.

Based on the sequence of the *crt* gene cluster in *P. psychrotolerans*, we designed two sets of primers and successfully amplified a ~ 9.4-kb carotenoid (*Pscrt*) biosynthetic gene cluster from *Pseudomonas* sp. 102515. The open reading frames (orfs) in this gene cluster were analyzed and annotated (Table 1), including *PscrtZ*, *PscrtB*, *PscrtI*, *PscrtY*, *PscrtX*, *Psidi*, *PscrtE,* and two orfs. Based on the predicted functions of these genes, we propose that this gene cluster is responsible for the biosynthesis of zeaxanthin diglucoside (Fig. 1).

**Table 1:** List of genes from the *Pscrt* biosynthetic gene cluster in *Pseudomonas* sp. 102515

Gene	Size (aa)	Function
*orf1*	314	YegS-like lipid kinase
*PscrtZ*	174	Beta-carotene hydroxylase
*orf2*	176	Gluconate 2-dehydrogenase subunit
*PscrtB*	311	15-cis-phytoene synthase
*PscrtI*	500	Phytoene desaturase
*PscrtY*	397	Lycopene cyclase
*PscrtX*	431	Zeaxanthin glycosyltransferase
*Psidi*	349	Isopentenyl-diphospahte delta-isomerase
*PscrtE*	293	Geranylgeranyl diphosphate synthase

### Functional characterization of two carotenoid biosynthetic genes from the endophyte

**Fig. 4:**
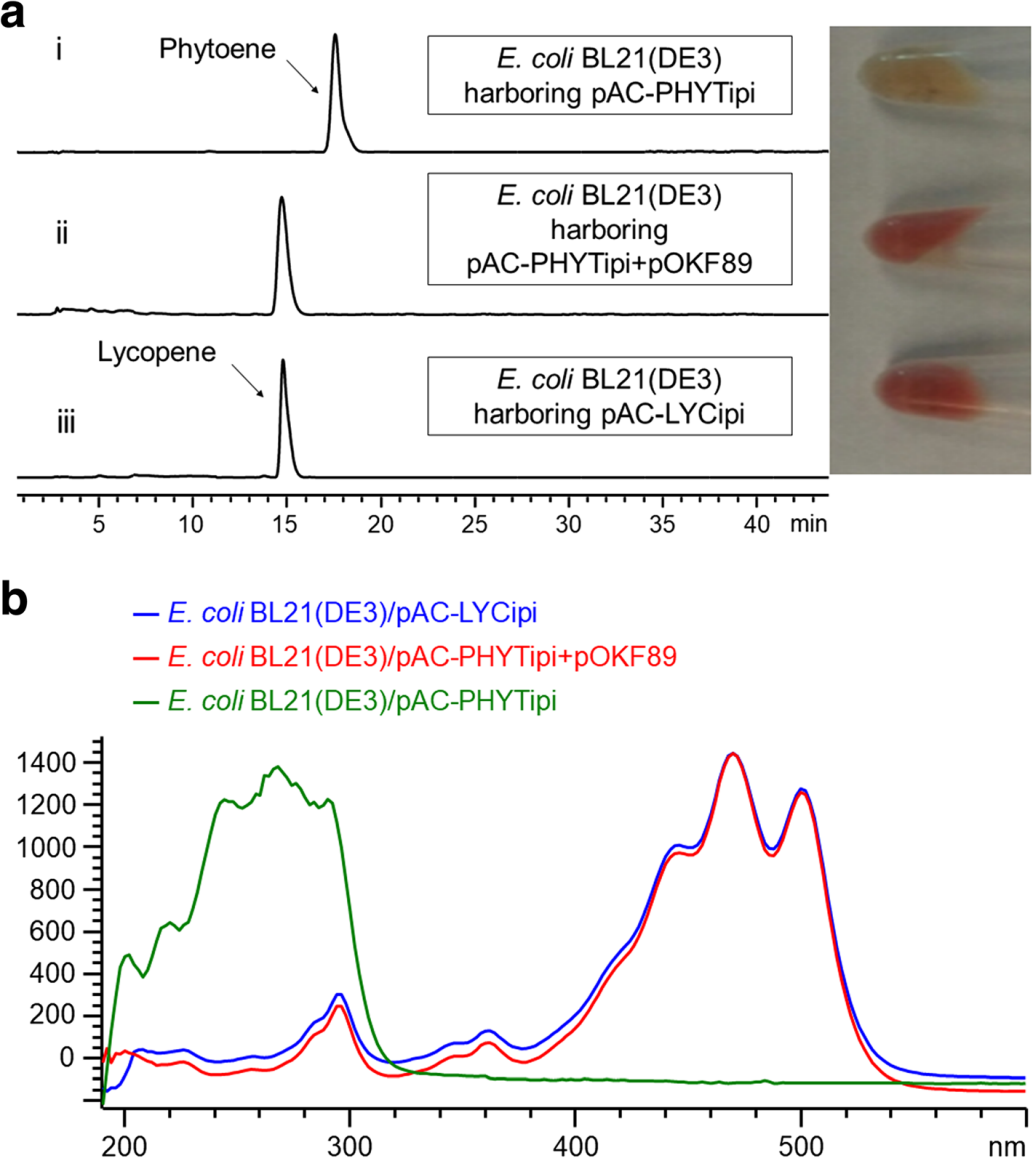
Functional identification of PsCrtI as a phytoene desaturase. (**a**) HPLC analysis of lycopene production through co-expression of PsCrtI with the phytoene biosynthetic enzymes. (i) *E. coli* BL21(DE3)/pAC-PHYTipi (negative control) producing phytoene (retention time: 17.5 min, 280 nm), (ii) *E. coli* BL21(DE3)/pAC-PHYTipi+pOKF89 (pAC-PHYTipi+PsCrtI) producing lycopene (retention time: 14.8 min, 460 nm), (iii) *E. coli* BL21(DE3)/pAC-LYCipi (positive control) producing lycopene (retention time: 14.8 min, 460 nm). Color change of harvested cells due to the co-expression of PsCrtI with pAC-PHYTipi in *E. coli* BL21(DE3) is shown on the right. (**b**) A comparison of the UV spectra of lycopene produced by *E. coli* BL21(DE3)/pAC-PHYTipi+pOKF89 with lycopene produced by the positive control and phytoene produced by the negative control

**Fig. 5:**
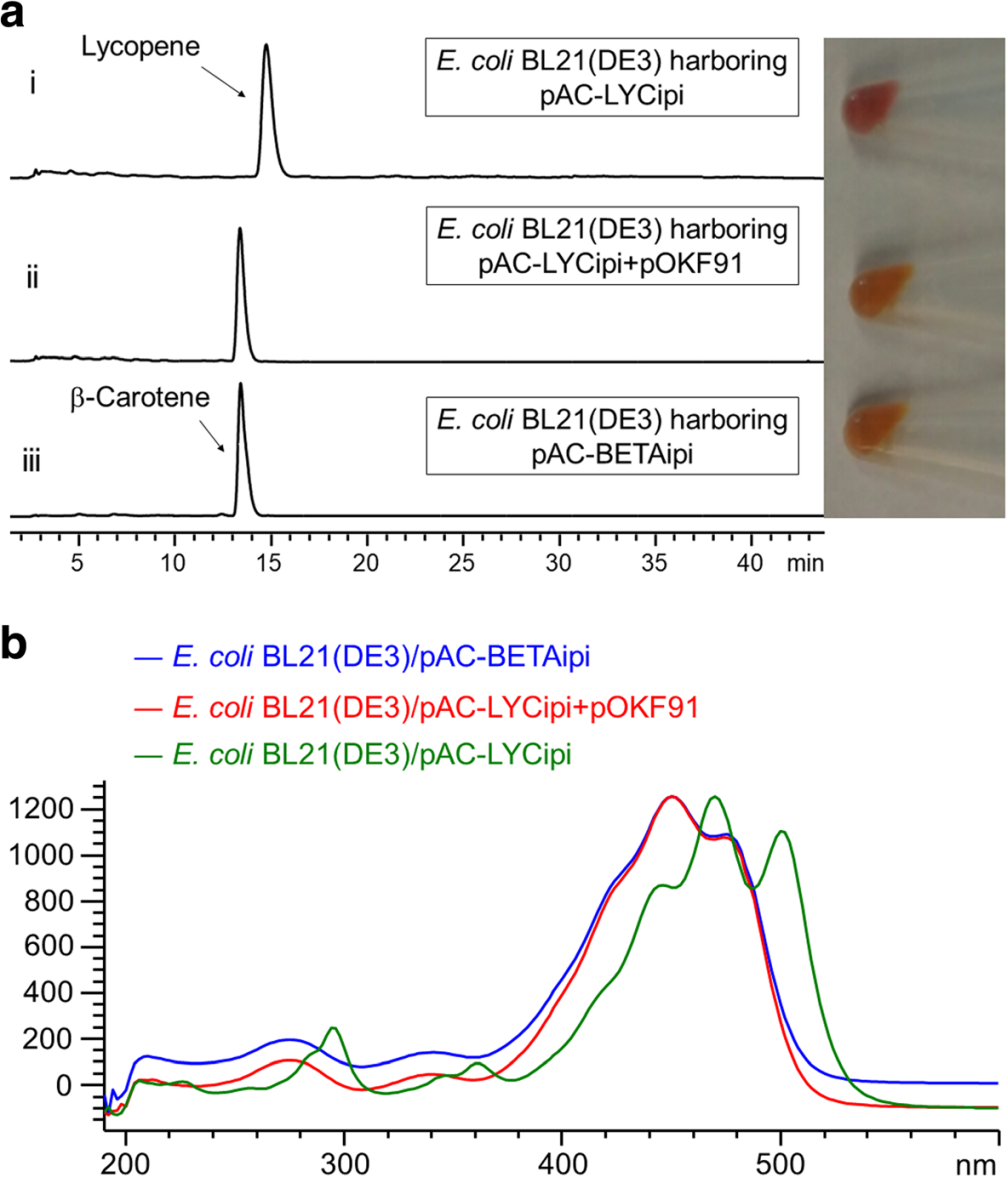
Functional identification of PsCrtY as a lycopene cyclase. (**a**) HPLC analysis (460 nm) of β-carotene production through co-expression of PsCrtY with the lycopene biosynthetic enzymes. (i) *E. coli* BL21(DE3)/pAC-LYCipi (negative control) producing lycopene (retention time: 14.8 min), (ii) *E. coli* BL21(DE3)/pAC-LYCipi+pOKF91 (pAC-LYCipi+PsCrtY) producing β-carotene (retention time: 13.3 min), (iii) *E. coli* BL21(DE3)/pAC-BETAipi (positive control) producing β-carotene (retention time: 13.3 min). Color change of harvested cells due to the co-expression of PsCrtY with pAC-LYCipi in *E. coli* BL21(DE3) is shown on the right. (**b**) A comparison of the UV spectra of β-carotene produced by *E. coli* BL21(DE3)/pAC-LYCipi+pOKF91 with β-carotene produced by the positive control and lycopene produced by the negative control

To characterize the functions of the carotenoid biosynthetic genes from *Pseudomonas* sp. 102515, it is necessary to develop an effective expression system for carotenoid biosynthetic enzymes in *E. coli*. We first ligated the *crtX* (zeaxanthin glucosyltransferase) gene from pAC-EHER into pET28a(+) to yield pOKF63. However, co-expression of pOKF63 and zeaxanthin-producing pAC-ZEAXipi in *E. coli* BL21(DE3) did not produce zeaxanthin diglucoside upon induction with 200 μM of IPTG. We next replaced the T7 promoter in pET28a(+) with a strong constitutive promoter J23119 and the B0034 ribosome binding site (pOKF72). Co-expression of pOKF72 and pAC-ZEAXipi in *E. coli* BL21(DE3) did yield zeaxanthin diglucoside, which was confirmed by HPLC through a comparison of the retention time and UV spectrum of the authentic sample obtained from *E. coli* BL21(DE3) harboring pAC-EHER (data not shown). We next used this system to test the functions of selected biosynthetic genes in the *Pscrt* gene cluster. PsCrtI and PsCrtY were chosen as their functions can be easily observed by the color change in the products. Co-expression of phytoene-producing plasmid pAC-PHYTipi with PsCrtI in *E. coli* BL21(DE3) led to the production of lycopene. We observed that the color of cell pellets changed from colorless (*E. coli* BL21(DE3)/pAC-PHYTipi) to red, as seen in Fig. 4a, indicating that a red compound was produced. For further confirmation, we compared the extract of this engineered strain with those of the negative (pAC-PHYTipi) and positive (pAC-LYCipi) controls by HPLC (Fig. 4a), which clearly revealed that lycopene was formed. This was supported by the UV spectra (Fig. 4b). This result confirmed that PsCrtI is a phytoene desaturase that converts phytoene to lycopene (Fig. 1). Similarly, we also confirmed the function of PsCrtY as a lycopene cyclase in *E. coli* BL21(DE3) by co-expressing pAC-LYCipi with PsCrtY. We observed the color change from red to yellow due to the conversion of lycopene into β-carotene by PsCrtY (Fig. 5a). Production of β-carotene was further confirmed by its retention time (Fig. 5)a and UV spectrum (Fig. 5b).

### Heterologous expression of the *Pscrt* gene cluster in *E. coli* BL21(DE3) and *Pseudomonas putida* KT2440

**Fig. 6:**
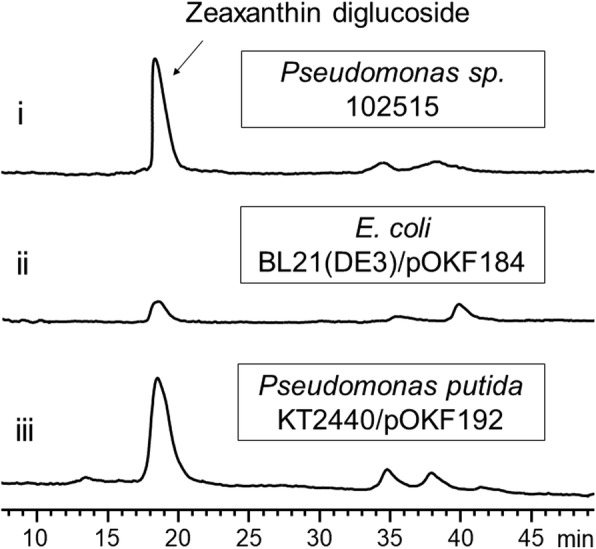
Heterologous reconstitution of the zeaxanthin diglucoside biosynthetic pathway in *E. coli* BL21(DE3) and *P. putida* KT2440. Shown are HPLC traces (460 nm) of the extracts of *Pseudomonas* sp. 102515 (i), *E. coli* BL21(DE3)/pOKF184 (ii), and *P. putida* KT2440/pOKF192 (iii)

Identification of PsCrtI and PsCrtY further suggested that this gene cluster is responsible for the biosynthesis of zeaxanthin diglucoside in *Pseudomonas* sp. 102515. Instead of characterizing each of the remaining genes in this gene cluster, we attempted to express the entire gene cluster in a heterologous host. We first transferred the *Pscrt* gene cluster into pET28a(+). Heterologous expression of the *Pscrt* gene cluster through the pET28a(+) expression system did not produce detectable amounts of zeaxanthin diglucoside. Then, we cloned the *Pscrt* gene cluster into a pACYC184-based expression system that has been previously used for the heterologous production of carotenoids in *E. coli* [28]. Expression of the *Pscrt* gene cluster in *E. coli* BL21(DE3) led to the production of zeaxanthin diglucoside (Fig. 6), which confirmed that this gene cluster is indeed responsible for the biosynthesis of this glycosylated carotenoid. However, the yield of zeaxanthin diglucoside was very low in *E. coli* BL21(DE3) as the cells only showed a slight yellow color. We figured that *P. putida* KT2440 might be a better host since it belongs to the genus of *Pseudomonas*. To this end, we cloned the *Pscrt* biosynthetic gene cluster into the pMIS1-mva vector, and expressed the resulting plasmid (pOKF192) in *P. putida* KT2440. Expression of this plasmid in *P. putida* KT2440 was deemed successful by observing the bright yellow color of the cells, and the formation of zeaxanthin diglucoside was verified by HPLC analysis (Fig. 6).

### Enhanced production of zeaxanthin diglucoside in *Pseudomonas* sp. 102515

**Fig. 7:**
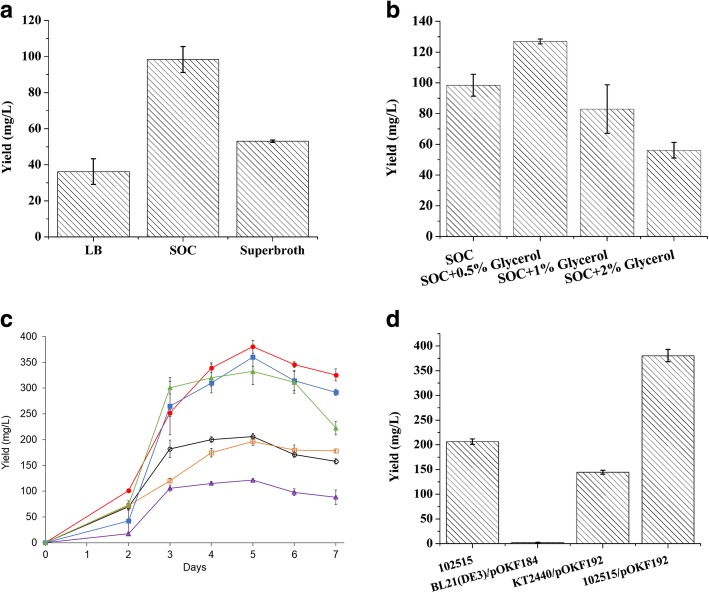
Optimization of zeaxanthin diglucoside production in *Pseudomonas* sp. 102515. (**a**) The effects of culture media on the production of zeaxanthin diglucoside. (**b**) The effects of supplementation of glycerol on the production of zeaxanthin diglucoside in the flasks. (**c**) The effects of cultivation temperature and time on the production of zeaxanthin diglucoside by *Pseudomonas* sp. 102515 and *Pseudomonas* sp. 102515/pOKFF192. Cells were grown in 50 mL of SOC medium supplemented with 0.5% glycerol. Black unfilled circle: 102515 at 18 °C; orange square: 102515 at 23 °C; purple unfilled triangle: 102515 at 28 °C; red filled circle: 102515/pOKF192 at 18 °C; blue filled square: 102515/pOKF192 at 23 °C; green filled triangle: 102515/pOKF192 at 28 °C. (**d**) A comparison of the production yield of zeaxanthin diglucoside by *Pseudomonas* sp. 102515 and three engineered strains

To improve the production of zeaxanthin diglucoside in *Pseudomonas* sp. 102515, we next attempted to optimize the culture conditions. We used a UV spectrophotometer to measure the amount of zeaxanthin diglucoside in the extracts at 456 nm, which is the wavelength of maximum absorbance for zeaxanthin diglucoside as reported in the literature [29]. Using the standard curve prepared with purified zeaxanthin diglucoside (Additional file 1: Figure S1), we calculated the yields from the crude extracts of each sample. Seven different media with 3 replicates were tested for the production of zeaxanthin diglucoside by *Pseudomonas* sp. 102515 in culture tubes. LB, SOC and superbroth media showed better yields compared to the other media (Additional file 1: Figure S2). We then tested LB, SOC and superbroth media in 50-mL flasks, which showed even better yields of zeaxanthin diglucoside than the tubes. As shown in Fig. 7a, SOC exhibited the best yield (98 ± 7 mg/L) among these three media.

Glycerol was previously reported to enhance the production of carotenoid [30]. Therefore, we tested the effects of glycerol on the production of zeaxanthin diglucoside by this endophyte in flasks at different concentrations (0.5, 1 and 2% final concentration). Supplementation of 0.5% glycerol (final concentration) into SOC medium increased the yield of zeaxanthin diglucoside to 127 ± 2 mg/ L after 3 days at 28 °C, representing a 30% increase in the yield (Fig. 7b). We next examined how different cultivation times and temperatures (18 °C, 23 °C, 28 °C and 37 °C) affect the yield of zeaxanthin diglucoside in SOC medium with 0.5% glycerol. The cultivation temperature of 37 °C resulted in extremely low yields (data not shown). Among the tested temperatures, 18 °C worked best and the yield reached 206 ± 6 mg/L in SOC medium after 5 days of cultivation (Fig. 7c). Under these conditions, we also tested the yields of zeaxanthin diglucoside in the engineered strains of *E. coli* BL21(DE3) and *P. putida* KT2440. Consistent with the cell colors, *E. coli* BL21(DE3) only produced 2 mg/L zeaxanthin diglucoside, while *P. putida* KT2440/pOKF192 generated the product in a much higher yield (121 ± 6 mg/L). However, since this yield is still lower than the wild type (206 mg/L), the latter is a better starting strain for further improvements. We successfully engineered *Pseudomonas* sp. 102515 by introducing pOKF192 into this strain, which allows this endophyte to have another copy of the *Pscrt* gene cluster. Expression of this plasmid further improved the yield to 380 ± 12 mg/L after 5 days of incubation at 18 °C (Fig. 7c and d).

## Discussion

Carotenoids have a variety of health-benefiting activities. They are also an important dietary source of vitamin A [31], thus representing a group of industrially important natural products. Carotenoids are considered to protect cells from the damaging effects of reactive oxygen species (ROS), which are formed by normal metabolic activities and lifestyle factors such as diet, smoking and exercise. Carotenoids might potentially diminish the destructive effects of ROS such as superoxide (O_2_^−^), hydrogen peroxide (H_2_O_2_), singlet oxygen (^1^O_2_) and hydroxyl radical (OH) [32, 33]. In specific, ^1^O_2_ is a product in both biochemical and photochemical systems that is responsible for the cell destruction caused by light and certain photosensitizers. The number of conjugated double bands in the carotenoids is a significant factor for the ^1^O_2_ quenching activity [34, 35]. Addition of the glucose moiety was found to enhance the ^1^O_2_ quenching activity of carotenoids. For example, the ^1^O_2_ quenching activity of a series of carotenoids were tested in *E. coli*. The *k*_q_ value of zeaxanthin diglucoside was 3.5 times higher than that of zeaxanthin [33]. Similarly, the viability (38.7%) of zeaxanthin-diglucoside-producing strain of *E. coli* was higher than that (25.7%) of zeaxanthin-producing strain in the ^1^O_2_ generation medium [33], further indicating that zeaxanthin diglucoside has better protecting effects than its aglycone.

Carotenoids such as lycopene, lutein, β-carotene, astaxanthin, and zeaxanthin are quite common in nature. However, glycosylated carotenoids such as dihydroxylycopene diglucoside, adonixanthin diglucoside and zeaxanthin diglucoside are relatively rare [36–39]. Glycosylated carotenoids can be potent antioxidant agents with protective properties against photooxidative damages from ROS and visible light [40, 41]. For instance, a novel glycosylated carotenoid, caloxanthin 3′-β-D-glucoside, was reported to have a potent ^1^O_2_ quenching activity with an IC_50_ of 19 μM [42]. Moreover, Tatsuzawa et al. reported that the viability of zeaxanthin diglucoside producing *E. coli* cells is higher in an ^1^O_2_ generation mixture compared to the cells that produce other carotenoids such as zeaxanthin, astaxanthin, β-carotene, and canthaxanthin [33]. Glycosylated carotenoids are also reported to stabilize the membrane through integrating within the lipid membrane due to their polar functional groups [40, 43]. Synthetic coloring agents have the negative effects on human health and thus there is an increasing consumer demand for natural and health-promoting food ingredients. In addition to their promising biological activities, such as antioxidant, anticancer, and anti-inflammatory properties, glycosylated carotenoids can be used in a wide variety of applications, ranging from food colorants and feed supplements to nutritional and cosmetics purposes [15]. Typically, carotenoids are poorly soluble in water. By contrast, glycosylated carotenoids were found to have a much higher water solubility than the corresponding carotenoid aglycones while maintaining similar biological activities. For instance, the water solubility of zeaxanthin diglucoside is more than 60-fold higher than that of zeaxanthin, which makes the use of this carotenoid in the water environment much easier [44]. Therefore, engineered production of glycosylated carotenoids including zeaxanthin diglucoside that are rare in nature is of great importance.

In this study, we isolated an endophytic bacterium *Pseudomonas* sp. 102515 from the leaves of *T. chinensis*, which was found to produce zeaxanthin diglucoside as a major metabolite. BLAST and phylogenetic analyses (Fig. 2) revealed that our endophytic isolate is a *Pseudomonas* strain. *Pseudomonas* species have been isolated from diverse sources including marine, freshwater, animals and plants [45–48]. Certain *Pseudomonas* strains, such as fluorescent *Pseudomonads*, are predominantly found in the rhizosphere and have been reported to move from rhizosphere to aerial plant tissue as in the case of *P. aeurofaciens* [49]. *Pseudomonas* species were also reported as endophytes in the literature [50, 51]. For instance, *P. stutzeri* A15 is an endophytic nitrogen-fixing bacterium isolated from paddy rice [52]. *P. fluorescens* was also reported to be an endophyte with beneficial interaction with plants [53]. The plant-endophyte interactions have not been fully understood. Many endophytes not only have beneficial effects on their hosts, but also plays a significant role in plant physiology. For example, some endophytic bacteria were reported to provide phytohormones, low-molecular compounds or enzymes to the host plants that led to the enhanced plant growth [54–56]. Endophytic bacteria also provide an alternative way to manage plant pathogens as a promising biocontrol agent through various ways, such as releasing antimicrobial substances, producing siderophores, and inducing the systemic resistance to pathogens [57–59]. For instance, biological control of *P. syringae* pv. *actinidiae*, the causal agent of bacterial canker of kiwifruit, was achieved by using an endophytic bacterium isolated from a medicinal plant [60].

To better understand *Pseudomonas* sp. 102515, we looked into the closely related strains, *P. psychrotolerans* and *P. oryzihabitans*. Both strains were reported as yellow-pigmented gram-negative bacteria and isolated from different sources including clinical samples, copper coins, diseased rice and rice seeds [61–65]. *P. oryzihabitans* was previously isolated from *Hibiscus rosasinensis* as an endophyte [66]. *P. psychrotolerans* was found to be an endosymbiotic bacterium from diseased rice and rice seeds in two different studies [61, 65]. The later endophytic bacterium was reported to enhance the plant growth due to potential nitrogen fixing characteristics of the turnerbactin (*tnb*) biosynthetic gene cluster, which is responsible for the biosynthesis of turnerbactin, a tricatecholate siderophore. Plants infected with *P. psychrotolerans* PRS08–11306 showed enhanced growth [25, 65]. We were able to amplify one of the key genes, *tnbA*, suggesting the existence of a *tnb* gene cluster in *Pseudomonas* sp. 102515. Thus, this endophyte may have potential as plant growth-promoting bacterium for agricultural applications.

**Fig. 8:**
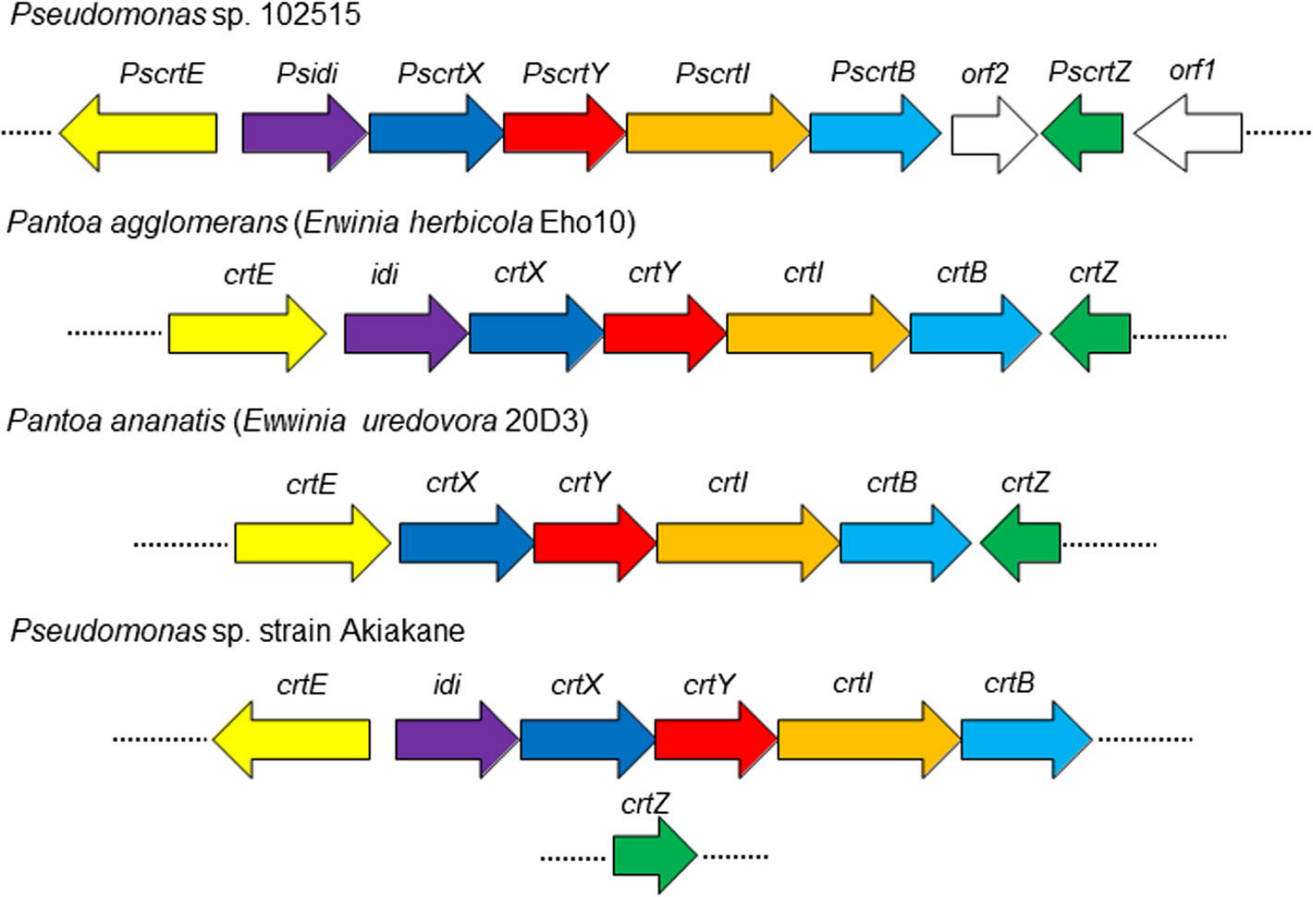
Organization of several carotenoid biosynthetic gene clusters in γ-proteobacteria. GenBank accession numbers: M87280 for *Pantoa agglomerans*, CP001875 for *Pantoa ananatis*, and LC317091 and LC317092 for *Pseudomonas* sp. strain Akiakane [48]

In addition to the *tnb* gene cluster, we found a complete carotenoid (*Pscrt*) biosynthetic gene cluster in *Pseudomonas* sp. 102515, which contains a series of carotenoid biosynthetic genes (Table 1**)**. However, there are some differences in the organization of the genes between the *Pscrt* gene cluster and other reported ones (Fig. 8). One obvious difference is the additional non-carotenoid gene (*orf2*) encoding for gluconate 2-dehydrogenase, which has not been reported in a carotenoid biosynthetic gene cluster to our knowledge. The role of this gene remains to be characterized. Typically, all genes in a *crt* biosynthetic gene cluster except *crtZ* have the same direction and are controlled by the same promoter in many γ-proteobacteria [27]. However, *PscrtE* also has a different direction compared to most of the genes in the *Pscrt* gene cluster and is controlled by its own promoter (Fig. 8). The similar organization of *crtE* in the gene cluster was also reported in *Pseudomonas* sp. strain Akiakane isolated from the excrement of autumn darker, yet its *crtZ* gene in that gene cluster is located differently than ours and overlapped with the sequences of *crtX* and *crtY* as shown in Fig. 8 [48]. We analyzed the functions of two *Pscrt* genes amplified from *Pseudomonas* sp. 102515 to confirm their functions through co-expression with heterologous carotenoid-producing genes in *E. coli* BL21(DE3) [67, 68]. PsCrtI was confirmed to be a phytoene desaturase and PsCrtY is a lycopene cyclase. The function of the *Pscrt* gene cluster was characterized by expression of the entire biosynthetic gene cluster in two heterologous hosts, *E. coli* BL21(DE3) and *P. putida* KT2440, both yielding zeaxanthin diglucoside (Fig. 6). This heterologous expression strategy not only allows the functional confirmation of this gene cluster, but also provides an alternative way to produce zeaxanthin diglucoside. However, the yield of zeaxanthin diglucosde in *E. coli* was very low compared to *Pseudomonas* sp. 102515, likely due to the low efficiency of the native promoter in this host. Sometimes a native promoter might not work well in a heterologous host. For instance, expression of the two *phaC* genes from *P. putida* were tested in *E. coli* under the control of a native promoter or an external promoter. They were only expressed in *E. coli* with the external promoter [69]. In another study, the levansucrase genes from *P. syringae* pv. *glycinea* PG4180 and *P. syringae* pv. *phaseolicola* NCPPB 1321 were cloned with the native promoters, yet the expression of levansucrase was only achieved upon the use of a *lac* promoter [70]. Therefore, we decided to use external promoters, in particular constitutive promoters, for heterologous expression of the *Pscrt* biosynthetic gene cluster from *Pseudomonas* sp. 102515 in *E. coli*. This approach allowed us to successfully reconstitute the production of zeaxanthin diglucoside in *E. coli*. Another approach we used was to choose another host microorganism. Since our isolate is a *Pseudomonas* strain, we proposed that the *Pscrt* gene cluster might be expressed better in *P. putida* KT2440 than *E. coli*. *P. putida* KT2440 was previously used as a heterologous host for the production of natural products including zeaxanthin [71, 72]. Our results confirmed that expression of the *Pscrt* gene cluster in *P. putida* KT2440 resulted in a much higher yield of zeaxanthin diglucoside than in *E. coli* (Fig. 7d).

A common strategy to improve the yield is the optimization of culture conditions [73]. We used LB medium for the isolation of *Pseudomonas* sp. 102515 and the initial product analysis of this endophyte. However, the nutrients in different media can affect the production of zeaxanthin diglucoside. A scanning of 7 different media (Additional file 1: Table S1) in culture tubes showed that LB, superbroth and SOC media had better yields ranging from 13 to 15 mg/L (Additional file 1: Figure S2). We chose SOC medium for further optimization studies due to its higher yield in flasks (Fig. 7a) and found that 0.5% glycerol supplementation further increased the yield to 127 ± 2 mg/L (Fig. 7b), which is consistent with a previous work on the effect of glycerol on the production of carotenoids [30]. It was found that supplementation of higher concentrations of glycerol decreased the titer of zeaxanthin diglucoside (Fig. 7B). This might be due to the inhibitory effect of 3-hydroxypropionaldehyde, a metabolite of glycerol. This compound was previously reported to inhibit the growth of *Enterobacter agglomerans*, which, similar to our strain, is a gram-negative bacterium with the carotenoid biosynthesis capability [74]. The effects of cultivation temperature and time were also examined. We originally used 28 °C as cultivation temperature as reported in the literature for the production of carotenoids [28, 67, 68, 71]. However, a comparison of four different temperatures revealed that 18 °C was the best cultivation temperature for the production of zeaxanthin diglucoside in *Pseudomonas* sp. 102515. The yield reached 145 ± 5 mg/L in 50 mL of SOC medium. We then cultivated this endophyte in SOC medium supplemented with 0.5% glycerol (final concentration) at 18 °C for a week to find out the best fermentation time. Based on the time course analysis, the yield reached the highest (206 ± 6 mg/L) after 5 days of cultivation. Low cultivation temperature is an economic burden for industrial production although it often favors the expression of enzymes [75]. Therefore, we cultivated *Pseudomonas* sp. 102515 in 50 mL of SOC medium supplemented with 0.5% glycerol at 23 °C for a week (Fig. 7c). After 5 days of cultivation, we obtained the yield of 197 ± 8 mg/L that is slightly lower than but comparable to the yield at 18 °C. Thus, 23 °C can be used for industrial production of zeaxanthin diglucoside with *Pseudomonas* sp. 102515. To further improve the yield, we introduced pOKF192 carrying the *Pscrt* gene cluster into *Pseudomonas* sp. 102515. With this additional copy of the *Pscrt* gene cluster, *Pseudomonas* sp. 102515/pOKF192 produced zeaxanthin diglucoside at 380 ± 12 mg/L, which is 85% higher than the parent strain and represents the highest yield of this promising antioxidant (Fig. 7c and d). Similar to *Pseudomonas* sp. 102515, this engineered strain at 23 °C led to a slightly lower but comparable yield than 18 °C (Fig. 7c).

## Conclusions

In conclusion, we isolated a carotenoid-producing endophytic *Pseudomonas* strain from the yew tree, which can potentially offer an alternative way to produce zeaxanthin diglucoside. A complete *Pscrt* gene cluster was discovered in this endophyte, from which the functions of two *Pscrt* genes were characterized in *E. coli* BL21(DE3). We cloned the whole *Pscrt* gene cluster and successfully expressed it in the heterologous hosts, *E. coli* BL21(DE3) and *P. putida* KT2440. PCR analysis also showed that a turnerbactin biosynthetic gene cluster exists in the endophytic strain, which renders our isolate a potential plant-growth-promoting bacterium for agricultural applications. Through fermentation and genetic engineering, we increased the yield of zeaxanthin diglucoside to 380 ± 12 mg/L. This engineered strain represents a great host for industrial production of this promising glycosylated carotenoid.

## Methods

### Bacterial strains, media and culture conditions

*E. coli* XL1-Blue, which was routinely grown in LB (Luria-Bertani) medium at 37 °C, was used for general genetic manipulations. *Pseudomonas* sp. 102515 was grown in LB overnight for genomic DNA extraction. LB, SOB, SOC, 2 × YT, TB, 2 × TB and superbroth media (Recipes for media are in Additional file 1: Table S1) were tested for the production of zeaxanthin diglucoside by *Pseudomonas* sp. 102515. *E. coli* BL21(DE3) and *P. putida* KT2440 were utilized for heterologous expression and co-expression studies. All wild type and engineered bacterial strains are listed in Table 2. Chloramphenicol (25 μg/mL), ampicillin (50 μg/mL) and kanamycin (50 and 30 μg/mL) were supplemented when appropriate. The concentrations of kanamycin for engineered *E. coli* and *Pseudomonas* strains are 50 and 30 μg/mL, respectively.

**Table 2:** Strains used in this study

Strain	Description	Source
*E. coli* XL1-Blue	endA1 gyrA96(nal^R^) thi-1 recA1 relA1 lac glnV44 F′[::Tn10 proAB^+^ lacI^q^ Δ (lacZ)M15] hsdR17(r_K_^−^ m_K_^+^)	Agilent Technologies
*E. coli* BL21(DE3)	BF^−^ ompT gal dcm lon hsdS_B_ (r_B_^−^m_B_^−^) λ (DE3 [lacI lacUV5-T7p07 ind1 sam7 nin5]) [malB^+^]_K-12_(λ^S^)	Novagen
*Pseudomona*s sp. 102515	Isolated endophytic strain from *Taxus chinensis*	This study
*Pseudomonas putida* KT2440	Heterologous host for expression of the *Pscrt* biosynthetic gene cluster	[76]
*E. coli* BL21(DE3)/pAC-PHYTipi	*E. coli* BL21(DE3) expressing the phytoene biosynthetic genes from pAC-PHYTipi (phytoene producing)	This study
*E. coli* BL21(DE3)/pAC-pHYTipi+pOKF89	*E. coli* BL21(DE3) expressing the phytoene biosynthetic genes from pAC-PHYTipi with *PscrtI* (lycopene producing)	This study
*E. coli* BL21(DE3)/pAC-LYCipi	*E. coli* BL21(DE3) expressing the lycopene biosynthetic genes from pAC-LYCipi (lycopene producing)	This study
*E. coli* BL21(DE3)/pAC-LYCipi+pOKF91	*E. coli* BL21(DE3) expressing the lycopene biosynthetic genes from pAC-LYCipi with *PscrtY* (β-carotene producing)	This study
*E. coli* BL21(DE3)/pAC-BETAipi	*E. coli* BL21(DE3) expressing the β-carotene biosynthetic genes from pAC-BETAipi (β-carotene producing)	This study
*E. coli* BL21(DE3)/pAC-ZEAXipi+pOKF72	*E. coli* BL21(DE3) expressing the β-carotene biosynthetic genes from pAC-ZEAXipi with CrtX (zeaxanthin diglucoside producing)	This study
*E. coli* BL21(DE3)/pAC-EHER	*E. coli* BL21(DE3) expressing the zeaxanthin diglucoside biosynthetic genes from pAC-EHER (zeaxanthin diglucoside producing)	This study
*E. coli* BL21(DE3)/pOKF184	*E. coli* BL21(DE3) expressing the *Pscrt* biosynthetic gene cluster from *Pseudomonas* sp. 102515	This study
*Pseudomonas putida* KT2440/pOKF192	*Pseudomonas putida* KT2440 expressing the *Pscrt* biosynthetic gene cluster from *Pseudomonas* sp. 102515	This study
*Pseudomonas* sp. 102515/ pOKF192	Engineered *Pseudomonas* sp. 102515 strain with an additional *Pscrt* biosynthetic gene cluster in pMIS1-mva	This study

### Isolation of endophytic bacteria from *T. chinensis*

The leaves were collected from a cultivated shrub of *T. chinensis* on the Logan campus of Utah State University (Latitude: 41.7424, Longitude: − 111.8080). A voucher specimen is deposited in the Intermountain herbarium, accession number UTC00282046. The collected leaves were thoroughly washed in running water. The disinfection and isolation were performed according to de Oliveira Costa et al. with minor modifications [77]. Briefly, the leaves were disinfected superficially through dipping into 70% ethanol for 2 min 3 times, followed by rinsing with 70% ethanol 3 times. Then, the leaves were dipped into sterile distilled water for a few minutes and rinsed with sterile distilled water. This process was repeated 3 times. To confirm the disinfection protocol, aliquots of the sterile water used in the final rinse were plated in LB plates at 28 °C for 7 days and the plates are examined for the presence or absence of any microbial colonies.

The disinfected leaves were grounded with 6 mL of an aqueous NaCl solution (0.85%) using a sterile mortar and pestle. The tissue extract was subsequently incubated at 28 °C for 3 h to allow the complete release of endophytic microorganisms from the host tissue. For the isolation of endophytic bacteria, the tissue extract was diluted with the NaCl solution and plated on LB plates with different dilutions. The plates were incubated for around a week at 28 °C. Promising colonies were selected and streaked on fresh LB plates for the isolation of promising bacteria. In particular, we picked the pigment-producing colonies in an attempt to isolate a carotenoid-producing endophyte.

### PCR and general genetic manipulations

Standard molecular biology protocols were performed as previously described [78]. Genomic DNA of *Pseudomonas* sp. 102515 was extracted with a ZR Fungal/Bacterial DNA Miniprep Kit. Plasmid DNA extraction from *E. coli* cells was performed using a Thermo Scientific GeneJET Plasmid Miniprep Kit. PCR reactions were performed with an Arktik™ Thermal Cycler using Phusion DNA polymerase. Primers were ordered from Thermo Fisher Scientific and dissolved in TE buffer to the final concentration of 100 ng/mL.

### Identification and phylogenetic analysis of the endophytic bacterium

The partial 16S rRNA gene fragment was PCR amplified from the genomic DNA of *Pseudomonas* sp. 102515 using the 16S universal primers of GGCTACCTTGTTACGACTTC and AGTTTGATCCTGGCTCAG [79]. The PCR product was sequenced by Sanger’s method. The 16S rDNA sequence was deposited into GenBank under the accession number of MK610450. BLAST analysis of this 16S rDNA sequence was performed using the 16S ribosomal RNA sequences database as the reference. Using the 16S rRNA sequences of the related bacteria from BLAST analysis, we created a phylogenetic tree by the neighbor-joining method using an online platform (https://itol.embl.de/) [80]. For physiological characteristics, scanning electron microscopy images were taken after performing the fixation of the sample [81].

### Amplification and annotation of the *Pscrt* biosynthetic gene cluster

The colonies of *Pseudomonas* sp. 102515 on LB agar plates showed a yellow color. LC-MS analysis of the chloroform extract of the cells confirmed that zeaxanthin diglucoside was produced. To discover the carotenoid (*Pscrt*) biosynthetic gene cluster in *Pseudomonas* sp. 102515, we first analyzed the genome of *P. psychrotolerans* (GenBank accession number: NZ_CP018758), which is the closest relative to our strain based on the phylogenetic analysis. We used an online genome analysis platform, AntiSMASH, and found a complete *crt* biosynthetic gene cluster responsible for the biosynthesis of zeaxanthin diglucoside. We designed two sets of primers (primers 3–6, Additional file 1: Table S2) based on the DNA sequence of this *crt* gene cluster*.* Briefly, the whole *Pscrt* gene cluster was divided into two fragments, which were PCR amplified from the genomic DNA of *Pseudomonas* sp. 102515 using the primers (primers 3 and 4 for fragment A, and primers 5 and 6 for fragment B) listed in Additional file 1: Table S2. Fragments A and B were first ligated into pJET1.2, yielding pOKF163 and pOKF166 (Table 3), respectively. These two plasmids were sent out for DNA sequencing to obtain the whole DNA sequence of the *Pscrt* gene cluster using the walking primers (primes 7–16) listed in Additional file 1: Table S2. The sequence of the *Pscrt* biosynthetic gene cluster from *Pseudomonas* sp. 102515 was deposited into GenBank under the accession number of MK613929.

**Table 3:** Plasmids used in this study

Plasmid	Description	Source
pJET1.2	Cloning vector	Fermentas
pET28a(+)	*E. coli* expression plasmid, pBR322, copy number: ~ 40	Novagen
pACYC184	*E. coli* expression plasmid, p15A, copy number: 15	ATCC 37033
pAC-PHYTipi	Phytoene producing plasmid	Addgene plasmid #: 53283
pAC-LYCipi	Lycopene producing plasmid	Addgene plasmid #: 53279
pAC-BETAipi	β-carotene producing plasmid	Addgene plasmid #: 53277
pAC-ZEAXipi	Zeaxanthin producing plasmid	Addgene plasmid # 53287
pAC-EHER	Zeaxanthin diglucoside producing plasmid	Addgene plasmid #: 53262
pMIS1-mva-ges	Expression plasmid for *Pseudomonas putida* KT2440 that contains the mevalonic acid (MVA) pathway genes	[72]
pOKF55	*crtX* gene cloned from pAC-EHER in pJET1.2	This study
pOKF63	*crtX* gene in pET28a(+)	This study
pOKF69	*crtX* gene with the J23119 constitutive promoter and B0034 ribosome binding site in pJET1.2	This study
pOKF72	*crtX* gene with the J23119 constitutive promoter and B0034 ribosome binding site in pET28a(+)	This study
pOKF85	*PscrtI* gene cloned from *Pseudomonas* sp. 102515 in pJET1.2	This study
pOKF88	*PscrtY* gene cloned from *Pseudomonas* sp. 102515 in pJET1.2	This study
pOKF89	*PscrtI* gene with the J23119 constitutive promoter and B0034 ribosome binding site in pET28a(+)	This study
pOKF91	*PscrtY* gene with the J23119 constitutive promoter and B0034 ribosome binding site in pET28a(+)	This study
pOKF163	The *Pscrt* biosynthetic gene cluster fragment A in pJET1.2	This study
pOKF166	The *Pscrt* biosynthetic gene cluster fragment B in pJET1.2	This study
pOKF169	The *Pscrt* biosynthetic gene cluster in pJET1.2	This study
pOKF173	The *Pscrt* biosynthetic gene cluster in pET28a(+)	This study
pOKF184	The *Pscrt* biosynthetic gene cluster in pACYC184	This study
pOKF187	*tnbA* gene cloned from *Pseudomonas* sp. 102515 in pJET1.2	This study
pOKF192	The *Pscrt* biosynthetic gene cluster in pMIS1-mva	This study

### Construction of plasmids for functional characterization of PsCrtI and PsCrtY

We first constructed a pET28a(+)-based expression plasmid for the carotenoid biosynthetic genes in *E. coli*. *crtX* (glycosyltransferase) was PCR amplified using primers 17 and 18 (Additional file 1: Table S2) from pAC-EHER, which was a gift from Francis X Cunningham Jr. (Addgene plasmid # 53262). PCR product was directly ligated into the cloning vector, pJET1.2, yielding pOKF55 (Table 3), which was sequenced using the Sanger method. *crtX* was excised with *Nco*I and *Hind*III from pOKF55 and ligated into pET28a(+) between the same sites to yield pOKF63 (Table 3). pOKF63 and pAC-ZEAXipi (Addgene plasmid # 53287) were introduced into *E. coli* BL21(DE3) for co-expression. The engineered strain was grown in LB medium with 50 μg/mL kanamycin and induced with 200 μM of IPTG for product analysis.

To replace the T7 promoter in pET28a (+) with a strong constitutive promoter, J23119, we redesigned the forward primer for *crtX* by including the J23119 promoter and B0034 ribosome binding site before the start codon (Additional file 1: Table S2). We re-amplified *crtX* with J23119 and B0034 from pAC-EHER using this new forward primer and previous reverse primer (primers 19 and 20). The new PCR product was ligated into pJET1.2 (pOKF69, Table 3) and the sequence was confirmed by sequencing. Subsequently, *crtX* with J23119 and B0034 was excised from pOKF69 using *Bgl*II and *Hind*III, and then ligated into pET28a(+) between the same sites to yield pOKF72 (Table 3). pOKF72 was co-expressed with pAC-ZEAXipi (Addgene plasmid # 53287) in *E. coli* BL21(DE3), and the products of the engineered strain was analyzed by HPLC.

*Ps*c*rtI* and *PscrtY* were PCR amplified from the genomic DNA of *Pseudomonas* sp. 102515 using the primers (primers 21 and 22 for *PscrtI*, and primers 23 and 24 for *PscrtY*) listed in Additional file 1: Table S2 and were subsequently ligated into pJET1.2 to yield pOKF85 and pOKF88 (Table 3), respectively. After sequencing, *PscrtI* and *PscrtY* were transferred into pET28a(+) with J23119 and B0034 using the *Nde*I and *Hind*III sites to yield pOKF89 and pOKF91 (Table 3), respectively. PsCrtI (pOKF89) and PsCrtY (pOKF91) were separately co-expressed with pAC-PHYTipi (Addgene plasmid #: 53283) and pAC-LYCipi (Addgene plasmid #: 53279) in *E. coli* BL21(DE3), respectively.

### Heterologous expression of the *Pscrt* biosynthetic gene cluster from *Pseudomonas* sp. 102515 in *E. coli* BL21(DE3) and *P. putida* KT2440

For the heterologous expression of the *Pscrt* gene cluster from the endophytic strain, we first excised fragment A from pOKF163 using *Nhe*I and *Mfe*I and ligated it into pOKF166 that contains fragment B between the same sites to yield pOKF169 (Table 3), which harbors the entire *Pscrt* gene cluster in pJET1.2. We then ligated the *Pscrt* gene cluster excised from pOKF169 using *Spe*I and *Hind*III into pET28a(+) digested with the *Nhe*I and *Hind*III sites (pOKF173, Table 3). Since pET28a(+) has a relatively higher copy number, we decided to also ligate the *Pscrt* gene cluster from pOKF169 into pACYC184 between the *Xba*I and *Hind*III sites to yield pOKF184 (Table 3). The corresponding expression plasmids, including pOKF173 and pOKF184, were transferred into *E. coli* BL21(DE3) for heterologous expression of the *Pscrt* gene cluster.

We also constructed an expression plasmid for expression of the *Pscrt* gene cluster in *P. putida* KT2440. The pMIS1-mva-ges plasmid was generously provided by Jens Schrader and Josef Altenbuchner at Dechema-Forschungsinstitut and University of Stuttgart, respectively [71, 72]. The whole *Pscrt* gene cluster was excised from pOKF169 using *Spe*I and *Pme*I and ligated into pMIS1-mva-ges digested with *Avr*II and *Pme*I to yield pOKF192 (Table 3). pOKF192 was introduced into *P. putida* KT2440 by electroporation as described in the literature [82]. Briefly, *P. putida* KT2440 was grown until the OD_600_ reached 0.4 at 28 °C. The cells were immediately placed on ice and harvested by centrifugation. Then, the cells were washed three times with 300 mM sterile sucrose solution and re-suspended in 100 μL of 300 mM sucrose solution. The cells were mixed with pOKF192 and the mixture was transferred into a pre-chilled electroporation cuvette. After 10 min of incubation on ice, the following settings were used for electroporation: set voltage - 2.5 kV (12.5 kV/cm); capacitor - 25 μF. After electroporation, 900 μL of LB broth was immediately added to the cuvette. The mixture was then transferred into a culture tube, which was incubated at 28 °C with shaking (250 rpm) for 2 h. Finally, the cells were plated on LB agar plates with 30 μg/mL kanamycin and incubated at 28 °C. The transformants were picked and grown in LB with kanamycin for product analysis. The same electroporation protocol was followed to introduce pOKF192 into *Pseudomonas* sp. 102515.

### Discovery of a turnerbactin biosynthetic gene cluster in *Pseudomonas* sp. 102515 by PCR

To find out whether *Pseudomonas* sp. 102515 contains the turnerbaction biosynthetic genes, the *tnbA* gene was amplified from the genomic DNA of *Pseudomonas* sp. 102515 using the primers 25 and 26 listed in Additional file 1: Table S2. The PCR product was ligated into the pJET1.2 cloning vector to yield pOKF187 (Table 3), which was subsequently sent out for sequencing (Sequence S1).

### Optimization of carotenoid production, extraction and HPLC-MS analysis

For *Pseudomonas* sp. 102515, different culture media were tested to increase the yield of zeaxanthin diglucoside. Upon the determination of a suitable medium, we investigated the effect of glycerol supplementation as additional carbon source. Cultivation time and temperature were also tested for an improved production of carotenoids. We also analyzed the yield of zeaxanthin diglucoside in heterologous expression systems.

The wild type and engineered strains were typically cultured in 250-mL flasks containing 50 mL of medium. The cultures were centrifuged at 3500 rpm for 8 min to harvest the cells and the cell pellets were suspended in 50 mL of methanol. The cell suspension in methanol was sonicated for 5 min to extract carotenoids. After centrifugation at 3500 rpm for 8 min, the resulting extract was dried in vacuo, and the residues were redissolved in a dimethyl sulfoxide (DMSO)-methanol mixture (10%, v/v) for HPLC-MS analysis with a gradient mobile phase of acetonitrile-water from 50 to 90% over 45 min at 1 mL/min. Low-resolution ESI-MS spectra were obtained on an Agilent 6130 LC-MS to confirm the molecular weights of carotenoids. For co-expression experiments, we used a different HPLC condition in order for the better separation of phytoene, lycopene and beta-carotene. For this HPLC method, we used an isocratic elution system of methanol/tetrahydrofuran (6/4, v/v) at 1 mL/min over 45 min.

Zeaxanthin diglucoside was subsequently purified by HPLC with an Agilent Eclipse XDB-C18 column (5 μm, 250 mm × 4.6 mm) for the standard curve preparation. Purified zeaxanthin diglucoside powder was measured and dissolved in a specific volume of methanol to make a zeaxanthin diglucoside solution. Next, this zeaxanthin diglucoside solution was serially diluted. The absorbance value of each dilution was measured at 456 nm on a UV-Vis spectrophotometer, with methanol as the blank control. Based on the correlation between the absorbance value and concentration, a standard curve of zeaxanthin diglucoside was prepared to calculate the yields in the optimization experiments. All crude extracts were dissolved in methanol and their absorbance values at 456 nm were measured on the UV-Vis spectrophotometer. The yields were calculated based on the measured data and standard curve. All samples were done in three replicates.

## Additional file

Additional file 1: **Table S1.** Recipes of the media used in this study. **Table S2.** Oligonucleotides used in this study. **Figure S1.** Standard curve for zeaxanthin diglucoside using UV spectrophotometer at 456 nm. **Figure S2.** The effects of different media on the yield of zeaxanthin diglucoside in the culture tubes (5 mL). (DOCX 114 kb)

## Data Availability

The partial 16S rRNA gene sequence of *Pseudomonas* sp. 102515 is available to the public in NCBI under accession number MK610450. The sequence of the *Pscrt* biosynthetic gene cluster is available to the public in NCBI under accession number MK613929.

## Abbreviations

DMAPP: Dimethylallyl diphosphate
DMSO: Dimethyl sulfoxide
FPP: Farnesyl pyrophosphate
GGPP: Geranylgeranyl diphosphate
GPP: Geranyl diphosphate
IPP: Isopenthyl diphosphate
IPTG: Isopropyl β-D-1-thiogalactopyranoside
LC-MS: Liquid chromatography-mass spectrometry
ROS: Reactive oxygen species
rRNA: ribosomal RNA
