# Supplementary material for: Discovery and engineering of an endophytic Pseudomonas strain from Taxus chinensis for efficient production of zeaxanthin diglucoside
Source: J Biol Eng. 2019 Aug 1;13:66. doi: 10.1186/s13036-019-0196-x (PMC6676639; doi:10.1186/s13036-019-0196-x)
Supplement: Supplementary file 1 — Table S1. Recipes of the media used in this study. Table S2. Oligonucleotides used in this study. Figure S1. Standard curve for zeaxanthin diglucoside using UV spectrophotometer at 456 nm. Figure S2. The effects of different media on the yield of zeaxanthin diglucoside in the culture tubes (5 mL). (DOCX 114 kb) [file 13036_2019_196_MOESM1_ESM.docx]

**Supporting Information**

**for**

# Discovery and engineering of an endophytic *Pseudomonas* strain from *Taxus chinensis* for efficient production of zeaxanthin diglucoside

Ozkan Fidan^1^ and Jixun Zhan^1,2*^

^1^ Department of Biological Engineering, Utah State University, 4105 Old Main Hill, Logan, UT 84322-4105, USA

^2^ TCM and Ethnomedicine Innovation & Development Laboratory, School of Pharmacy, Hunan University of Chinese Medicine, Changsha, Hunan 410208, China

**Correspondence:** [jixun.zhan@usu.edu](mailto:jixun.zhan@usu.edu)

**Table S1.** Recipes of the media used in this study.

|  | **LB** | **SOB** | **SOC** | **2xYT** | **TB** | **2xTB** | **Superbroth** |
| --- | --- | --- | --- | --- | --- | --- | --- |
| Tyrptone (g/L) | 10 | 20 | 20 | 16 | 12 | 24 | 32 |
| Yeast extract (g/L) | 5 | 5 | 5 | 10 | 24 | 42 | 20 |
| NaCl (g/L) | 10 | 0.58 | 0.58 | 5 | - | - | 5 |
| Glycerol (ml/L) | - | - | - | - | 4 | 15 | - |
| Phosphate buffer (0.17 M KH_2_PO_4_, 0.72 M K_2_HPO_4_) (ml/L) | - | - | - | - | 100 | 100 | - |
| KCl (g/L) | - | 1.9 | 1,9 | - | - | - | - |
| MgCl_2_ (g/L) | - | 0.95 | 0.95 | - | - | - | - |
| MgSO_4_ (g/L) | - | 1.21 | 1.21 | - | - | - | - |
| Glucose (g/L) | - | - | 3.6 | - | - | - | - |

**Table S2.** Oligonucleotides used in this study.

| **Primer No.** | **Primers** | **Oligonucleotides** |
| --- | --- | --- |
| 1 | 16S rRNA universal primer 8F-F1 | ggctaccttgttacgacttc |
| 2 | 16S rRNA universal primer Rn1 | agtttgatcctggctcag |
| 3 | 10-25-15-BGC-A-SpeI-NdeI-fw | aactagtcatatgttggtggagatccgcgaggatcgcacc |
| 4 | 102515-A-NheI-rev | tggccggcggcctgctagccctgcgcctggcccaaac |
| 5 | 102515-B-NheI-fw | gccaggcgcagggctagcaggccgccggccaggccg |
| 6 | 10-25-15-BGC-B-PmeI-HindIII-rev2 | aagtttaaacaagctttcaacgaggacgaaagacgatat |
| 7 | BGC-A walker primer F1 | catgtgctctgtcccaccgccg |
| 8 | BGC-A walker primer R1 | agggcttcttccgcctgctcaa |
| 9 | BGC-A walker primer F2 | tccttcggtttcctgctggcgc |
| 10 | BGC-A walker primer R2 | accaggcccggctggacgagca |
| 11 | BGC-B walker primer F1 | cagttggcgacaggcctgggcg |
| 12 | BGC-B walker primer R1 | catcagcagcggacgcagcc |
| 13 | BGC-B walker primer F2 | cttcgtcttcgcctccttcgg |
| 14 | BGC-B walker primer R2 | agcgtgctcgatgtcgccggaa |
| 15 | Eho10-CrtX-NcoI-SmaI-fw | aaccatggcccgggatgagccattttgccatt |
| 16 | Eho10-CrtX-HindIII-rev | aaaagctttcacgatacgctctcactccctgctatggc |
| 17 | J23119-B0034-NcoI-CrtX-fw | agatctttgacagctagctcagtcctaggtataatgctagctactagagaaagaggagaaaccatggatgagccattttgccatt |
| 18 | Eho10-CrtX-HindIII-rev | aaaagctttcacgatacgctctcactccctgctatggc |
| 19 | 10-25-15-CrtI-NdeI-fw | aacatatgatgacccaaggtaacgcgcccaagcgggccat |
| 20 | 10-25-15-CrtI-HindIII-rev | aaaagctttcatggcttaccgtgctcctcgaggatgacgc |
| 21 | 10-25-15-CrtY-NdeI-fw | aacatatgatgcgccctgacctgctgatcgtcgggggt |
| 22 | 10-25-15-CrtY-HindIII-rev | aaaagctttcatggtgcttccttggactgagggggacgc |
| 23 | Eho10-CrtX-NcoI-SmaI-fw | aaccatggcccgggatgagccattttgccatt |
| 24 | Eho10-CrtX-HindIII-rev | aaaagctttcacgatacgctctcactccctgctatggc |
| 25 | 102515-TnbA-NdeI-fw | aacatatgacgcaattggatttcaccggcaagcg |
| 26 | 102515-TnbA-PmeI-HindIII-rev | aagtttaaacaagcttttacgccgccagggtcgcgc |

**Fig. S1.** Standard curve for zeaxanthin diglucoside using UV spectrophotometer at 456 nm.





**Fig. S2.** The effects of different media on the yield of zeaxanthin diglucoside in the culture tubes (5 mL).

Sequence S1. *Pseudomonas* sp. strain 102515 *tnbA* DNA sequence

ATGACGCAATTGGATTTCACCGGCAAGCGCGTCTGGGTGACCGGCGCGGGGCAGGGCATCGGCCTGCAGGTGGCGCGCCGCTTCCAGGCGGCGGGCGCCGAGGTGGTCGGTCTCGACCGGCGGTTCGCCGAGAGCAGCTATCCCTTTCGCATCCAGCTGCTGGATGTCGCCGATGCGGCCCAGGTGGAGGACGTCTGCCAGGCGCTGCTGGCGGAAGAACGCCGTCTGGACGTATTGGTCAACGGCGCCGGCGTGCTGCGCCTGGGCACCAGCGACAGCCTGAGCCTGGACGACTGGCAGGCGCTCTTGGACGTCAACGTCTCCGGCCCCTTCTACCTGTTCCGCCAACTGGTGCCGCTGTTCAAGGCCCAGGGCCAGGGCGCCATCGTCAACATCGCCTCCAACGCCGCCCATGTGCCGCGCCTGCAGATGGCCGGCTACTGCACCTCCAAGGCGGCGCTGGTGTCCCTCAGCCATTGCGTGGCCCTGGAACTGGCCGGGTCGGGGGTGCGCTGCAACGTCGTCTCGCCGGGCTCCACGGCGACCCCCATGCTGGCCGGCATGCTCGGCTCGCCCGAGGGTTATCAGCGCCTGGTAGACGGCCTGCCGGAGGATTTCAAGCTGGGCATCCCGCTGCGCAAGATCGCCCAGCCCGACGAGATCGCCAATACCGTGCTGTTCCTCGCCTCGGACCTGGCCAGCCATATCACCATGCAGGACATCGTGGTGGACGGCGGCGCGACCCTGGCGGCGTAA
